# Underreported Threat of Multidrug-Resistant Tuberculosis in Africa

**DOI:** 10.3201/eid1409.061524

**Published:** 2008-09

**Authors:** Yanis Ben Amor, Bennett Nemser, Angad Singh, Alyssa Sankin, Neil Schluger

**Affiliations:** Columbia University, New York, New York, USA

**Keywords:** Tuberculosis, Africa, multidrug resistance, perspective

## Abstract

Multidrug-resistant tuberculosis (MDR TB) in Africa may be more prevalent than previously appreciated. Factors leading to development of drug resistance need to be understood to develop appropriate control strategies for national programs. We gathered estimates of MDR TB rates for 39 of 46 countries in Africa. The relationship between MDR TB rates and independent factors was analyzed by using correlation and linear regression models. Our findings indicate that drug resistance surveys in Africa are critically needed. MDR TB rates must be assessed in countries without these surveys. In countries that have conducted a drug resistance survey, a new survey will determine evolution of drug resistance rates. We found no correlation between high MDR rates and TB incidence, HIV/TB co-infection rates, or year of introduction of rifampin. Results show that the retreatment failure rate was the most predictive indicator for MDR TB. Current category II drug regimens may increase MDR TB.

## Abstract 2

Identification of population-based factors should influence regional and national policy.

Global control of tuberculosis (TB) has been jeopardized by 2 major threats: HIV/AIDS and multidrug-resistant TB (MDR TB). MDR TB is defined as strains of *Mycobacterium tuberculosis* that are resistant to at least isoniazid and rifampin (*1*). Drug resistance has reached alarming levels with the emergence of strains that are virtually untreatable with existing drugs. The recent report of an outbreak of extensively drug-resistant TB (XDR TB) in South Africa (*2*), with its extremely high case-fatality rate, has drawn wide attention. However, the more general problem of MDR TB, with an estimated 450,000 cases worldwide annually, has been recognized since the first World Health Organization (WHO) global survey on drug resistance in the late 1990s (*3*).

According to the WHO Global Report on Anti-tuberculosis Drug Resistance in the World (*1*), MDR TB strains have emerged in all regions of the world. However, despite the dramatic increase in TB rates in Africa, MDR TB appears nearly absent from this continent, which, until recently, reported the lowest median levels of drug resistance.

Two explanations have been most commonly put forward to explain these reported low levels of MDR TB in Africa. The first explanation is the presence of well-functioning control programs in Africa. Eighty-nine percent of the population in the WHO-defined region of Africa is covered by directly observed therapy, short course (DOTS), which is similar to the global average (*4*). However, there is discordance between purported DOTS coverage and national TB program (NTP) efficacy. Each year, countries with the lowest case detection and cure rates are clustered in the WHO-defined Regional Office for Africa (AFRO) region (*4*). This suggests that NTPs in Africa are not performing better than their Eastern European or South American counterparts, where MDR TB rates have already reached alarming levels. The functional status of many programs in Africa is difficult to assess with certainty, and the high incidence rates on that continent indicate that programs may not be functioning well. Low case-detection rates alone may not lead to development of MDR TB. Other factors that might favor development of MDR TB include the availability of drugs on the open market and a private sector that delivers drugs to the population in an unregulated fashion.

The second explanation is the recent introduction of rifampin in Africa. It is often stated that because rifampin was only recently introduced in Africa on a large scale, there has been relatively little time for resistance to develop. However, development of rifampin resistance may be spurred by HIV infection, and such resistance appears to develop rapidly (*5*–*10*).

Our research described in this report indicates the possibility of a third and straightforward explanation: field results for Africa are still incomplete, despite the recent publication of the WHO Fourth Global Report (*1*). This report is the result of independent drug resistance surveys (DRSs) conducted throughout the world following strict guidelines. The number of countries participating in the overall survey was low, especially in the Africa region. Furthermore, one might speculate that those countries capable of providing DRS data may have been the ones most likely to have a well-functioning NTP, laboratory structures, and transport networks, which would bias overall reporting. However, even if this is not the case, overall low levels of reporting make findings of the report questionable.

Consequently, the low level of reported drug resistance in Africa may not be an accurate reflection of reality, and the limited evaluation may be responsible for this skewed portrayal. Lack of comprehensive national DRS data from all countries in Africa is a barrier to understanding the magnitude of prevalence and incidence of MDR. In light of the added threat of XDR TB within the African context of high HIV prevalence, a thorough assessment of TB drug resistance and evaluation of data-specific deficiencies is urgently needed.

Our report aims to provide a more accurate assessment of MDR TB in Africa by incorporating all the latest available data and published estimates. In addition to suggesting that the effect of MDR TB in Africa is higher than previously thought, our report explores trends and relationships among case detection, treatment success, retreatment failure, and HIV rates relative to MDR TB. We attempted to identify population-based factors associated with MDR TB. Given the limited healthcare funding and substantial incidence of HIV in Africa, even a relatively low but increasing tide of MDR TB can lead to disastrous consequences for this continent.

## Methods

### Search Strategy

PubMed and Medline databases were searched for reviews and original reports based on primary studies by using the keywords tuberculosis, *Mycobacterium tuberculosis*, multidrug-resistant, and Africa. English and French language articles were reviewed.

### Study Selection

Data were assembled into a comprehensive composite on the basis of several criteria. Published MDR TB rates from WHO were prioritized because of their reliability and because they are the result of DRSs conducted throughout the world following strict guidelines: new patients were clearly distinguished from those with previous TB treatment, and optimal laboratory performance was ensured and maintained through links with supranational reference laboratories.

Second, data on MDR TB were collected from peer-reviewed journals. Articles were considered only if specific criteria were met: 1) new patients needed to be distinguished from patients with prior TB treatment; 2) the number of confirmed TB cases needed to be statistically significant; and 3) the study needed to be nationwide or the population covered needed to be greater than one fourth of the total population.

For countries where data were unavailable through WHO reports or recent peer-reviewed journals, published formulaic estimates by Zignol et al. were used (*11*). The formula considers 9 independent variables considered likely to be associated with drug resistance and used in regression analyses. Resulting estimates compare favorably with known MDR TB values.

### Data Extraction

The following data were tabulated for all forms of TB for all countries under scrutiny. Presurvey and postsurvey variables were annual country-specific indicators averaged from 1995 to the year of the drug resistance survey (presurvey) and from year after drug resistance survey to 2005 (postsurvey). These variables included TB incidence rate, case detection rate, treatment rates (category I: cured, completed, failure, default and success), and retreatment rates (category II: cured, completed, failure, default, and success). Year 2005 variables were published MDR TB estimates for new cases of pulmonary TB, HIV prevalence among adults 15–49 years of age, TB-related death rate, HIV/TB co-infection rate, male-to-female ratio of case notification rate, and health funding per capita (US dollars) in 2002 obtained from the United Nations Development Program (http://hdr.undp.org/statistics/data/indic/indic_52_1_1.html).

Combined MDR TB rates were compiled for 39 of the 46 countries in the AFRO region. For 21 of those 39 countries, MDR TB rates were gathered from either WHO reports or peer-reviewed articles. For the remaining 18 countries, MDR TB rates were reported by using a formula described by Zignol et al. (*11*). No DRS data or formulas were available for 6 countries, Cape Verde, Comoros, Liberia, Mauritania, Sao Tome and Principe, and the Seychelles, which account for only 20,475 of the 2,528,915 TB cases in Africa (*4*). Additionally, the accuracy of annual data from Mauritius was deemed questionable and thus excluded from further analysis.

### Regression Analysis of Continuous Variables

The unadjusted associations between continuous independent variables and MDR TB rates were evaluated by using a linear correlation matrix. Independent variables highly (p<0.05) or marginally (p<0.10) predictive of the outcome in unadjusted models were included in further linear multivariable modeling. In addition, interactions between case detection, treatment failure, and retreatment failure rates were tested by using univariate linear regression analyses. All analyses were conducted in Stata Statistics version 9.2 (StataCorp, College Station, TX, USA).

### Analysis of Presurvey and Postsurvey Continuous Variables

The change between presurvey and postsurvey averages (average value from 1995 to year of drug resistance survey, and year of drug resistance survey to 2005, respectively) for each continuous independent variable was evaluated by using the nonparametric Wilcoxon signed rank test. Given the small sample size, these variables were not assumed to be normal. A change between presurvey and postsurvey was deemed statistically significant if the p value was <0.05 and marginally significant if <0.10.

### Categorical Trend Analysis

In addition to relationships between continuous MDR TB rates and independent factors, a categorical analysis of MDR TB was conducted. Countries were divided into 3 categories (tertiles) on the basis of MDR TB rates: (0.0–1.7, 1.8–2.1, and >2.2 defined as low, middle, and high MDR rates categories, respectively). For each TB-related indicator, a univariate trend across the 3 MDR TB categories (low, middle, and high) was assessed. Trend analysis was conducted by using the nptrend function in Stata Statistics version 9.2 (StataCorp), which performs a nonparametric trend test of rank sums across ordered groups. A trend was deemed statistically significant if the p value was <0.05 and marginally significant if <0.10.

## Results

### MDR TB Rates in Africa

Data on prevalence of MDR TB among all TB cases collected from various WHO publications and published peer-reviewed articles are shown in Table 1. The 39 countries considered in this study encompass ≈99% of total estimated incident or prevalent TB cases (all forms) in the AFRO region. The proportion of MDR TB among all TB cases varies from 5.8% in the Democratic Republic of Congo to virtually 0% in Kenya. The median MDR TB rate was ≈1.9% (Table 2).

**Table 1:** Prevalence of MDR TB among combined TB cases by country, Africa*

Country	MDR resistance value, % (95% CI)	Nationwide study	Year	Reference	References for other convenience sample surveys
Algeria	1.4† (0.5–2.7)	NA	NA	(*11*)	
Angola	2.1† (0.6–8.9)	NA	NA	(*11*)	
Benin	1.0† (0.2–3.9)	NA	NA	(*11*)	
Botswana	1.6‡ (0.8–1.9)	Countrywide survey	2002	(*1*)	(*17*,*18*)
Burkina Faso	2.6† (8.8–11.0)	NA	NA	(*11*)	(*19*)
Burundi	1.4§	Bujumbura survey	2006	(*20*)	
Cameroon	2.0† (0.6–8.9)	NA	NA	(*11*)	(*12*,*13*)
Central African Republic	2.2¶ (1.0–3.4)	Countryside survey	1998	(*1*)	(*21*)
Chad	1.9† (0.5–9.0)	NA		(*11*)	(*22*)
Congo, Brazzaville	1.8† (0.5–8.2)	NA	NA	(*11*)	
Côte d’Ivoire	5.4† (3.2–8.4)	NA	NA	(*11*)	(*23*)
Democratic Republic of Congo	5.8¶ (0.6–10.3)	Kinshasa survey	1999	(*1*)	
Equatorial Guinea	3.4§	5 of 18 survey districts	2004	(*24*)	
Eritrea	1.9† (0.5–9.0)	NA	NA	(*11*)	
Ethiopia	2.5¶	Countrywide survey	2005	(*1*)	(*25*–*27*)
Gabon	1.8† (0.6–8.1)	NA	NA	(*11*)	
Gambia	0.4¶ (0.0–1.4)	Countrywide survey	2000	(*28*)	
Ghana	1.9† (0.5–8.7)	NA	NA	(*11*)	
Guinea	2.1¶ (1.0–3.0)	Sentinel sites survey	1998	(*1*)	
Guinea-Bissau	2.6† (0.8–1.4)	NA	NA	(*11*)	
Kenya	0¶ (0.0–1.1)	Nearly countrywide survey	1995	(*1*)	(*29*–*31*)
Lesotho	1.6¶ (0.4–2.6)	Countrywide survey	1995	(*1*)	
Madagascar	0.7¶ (0.7–10.3)	Countrywide survey	2007	(*1*)	(*14*)
Malawi	1.9† (0.5–9.3)	NA	NA	(*11*)	(*15*,*16*)
Mali	1.5† (0.3–7.9)	NA	NA	(*11*)	
Mauritius	1.4† (0.4–6.4)	NA	NA	(*11*)	
Mozambique	3.5¶ (2.5–4.6)	Countrywide survey	1999	(*1*)	(*32*,*33*)
Namibia	1.5† (0.4–7.1)	NA	NA	(*11*)	
Niger	2.7† (0.8–11.5)	NA	NA	(*11*)	
Nigeria	2.0† (0.6–9.3)	NA	NA	(*11*)	
Rwanda	4.6	Countrywide survey	2005	(*34*)	
Senegal	4.3¶ (0.8–10.6)	Countrywide survey	2006	(*1*)	
Sierra Leone	3.1¶ (0.3–4.0)	Nearly countrywide survey	1997	(*1*)	
South Africa	3.1¶ (2.2–3.0)	Countrywide survey	2002	(*1*)	(*2*,*35*–*39*)
Swaziland	1.9¶ (0.5–3.1)	Countrywide survey	1995	(*1*)	
Togo	2.1† (0.6–9.5)	NA	NA	(*11*)	
Uganda	1¶ (0.1–1.6)	3 district surveys	1997	(*1*)	
Tanzania	1¶ (0.6–9.8)	Countrywide survey	2007	(*1*)	
Zambia	1.8¶ (0.8–3.1)	Countrywide survey	2000	(*1*)	
Zimbabwe	2.2¶ (1.3–4.0)	Nearly countrywide survey	1995	(*1*)	

*TB, tuberculosis; MDR TB, multidrug-resistant TB; CI, confidence interval; NA, not available. †Formulaic. ‡National Drug Resistance survey. §Convenience sample survey. ¶World Health Organization.

**Table 2:** Descriptive statistics for country-specific MDR rates and other TB-related factors, Africa*

Factor	Average, presurvey years (1995 to survey year)				Average, postsurvey years (year after survey to 2005)				Wilcoxon signed rank test†
	No., mean, median	Range	SD		No., mean, median	Range	SD		Z-score, p value‡
MDR rates	39, 2.21, 1.9	0.0–5.8	1.2						
Incidence rate/100,000/ y	39, 116, 109	22–228	40.6		33, 152, 150	25–308	70.2		–3.92, 0.0001§
Case detection rate (new ss+)	39, 47.2, 49.8	8.2–86.0	22.4		32, 55.5, 56.2	13.6–112.2	27.5		–1.52, 0.130
Treatment indicators									
Cured	36, 50.9, 52.3	17.1–74.6	14.2		32, 56.2, 59.0	16.6–78.4	14.5		–3.58, 0.0001§
Completed	39, 14.3, 12.4	3.1–47.4	9.8		32, 13.9, 11.1	1.3–38.5	8.9		1.27, 0.206
Died	36, 6.7, 6.1	2.6–19.5	3.3		32, 7.4, 7.2	0.7–17.1	3.5		–0.36, 0.721
Failed	36, 1.8, 1.6	0.0–7.8	1.5		32, 1.7, 1.3	0.2–5.3	1.2		–0.34, 0.738
Defaulted	36, 14.1, 13.2	4.4–41.4	6.8		32, 11.9, 10.9	2.6–39.7	7.4		2.48, 0.013§
Succeeded	36, 65.2, 67.0	33.0–83.5	10.3		32, 70.0, 70.5	37.1–90.3	10.3		–3.56, 0.0001§
Retreatment indicators									
Cured	35, 47.1, 48.0	11.1–71.3	16.2		29, 48.5, 52.0	2.9–72.4	17.3		–1.22, 0.221
Completed	35, 14.1, 12.7	0.0–46.6	8.7		29, 13.4, 9.8	0.5–40.8	10.0		1.49, 0.135
Died	35, 7.9, 7.7	0.0–22.7	4.1		29, 10.4, 10.1	1.6–21.4	4.5		–2.27, 0.023§
Failed	35, 3.6, 2.8	0.2–14.6	2.8		29, 3.2, 3.0	–8.9	2.2		0.18, 0.861
Defaulted	35, 14.6, 12.8	4.6–29.3	6.9		29, 11.9, 10.9	2.7–26.7	6.2		1.49, 0.135
Succeeded	35, 61.1, 62.6	30.3–81.3	13.1		29, 61.9, 64.6	23.1–81.8	14.2		0.18, 0.861
Year 2005 only variables									
Prevalence/100,000					38, 497, 513	55–936	178		
TB mortality rate/100,000/ y					39, 79, 73	2–304	48		
HIV/TB co-infection, %					39, 26.8, 19.0	0.5–75.0	20.5		
Male/female ratio: case notifications					36, 1.5, 1.5	0.7–2.6	0.4		
Health expenditures (US $ per capita)					39, 107, 51	15–689	131		

*MDR, multidrug resistance; TB, tuberculosis, SD, standard deviation; ss+, sputum sample positive. †Based on presurvey minus postsurvey values. A negative Z-score is indicative of an increase over time. ‡Marginally statistically significant trend (p<0.10). §Statistically significant trend (p<0.05).

The situation in Africa for MDR TB among all combined TB cases is depicted in a series of related maps (Figure). The first map is reproduced and translated from the WHO Third Global Report published in 2004 (*40*) (Figure, panel A). The coloring scheme was translated into the 3 categories used in this report, and the continent appears particularly devoid of MDR TB.

**Figure:**
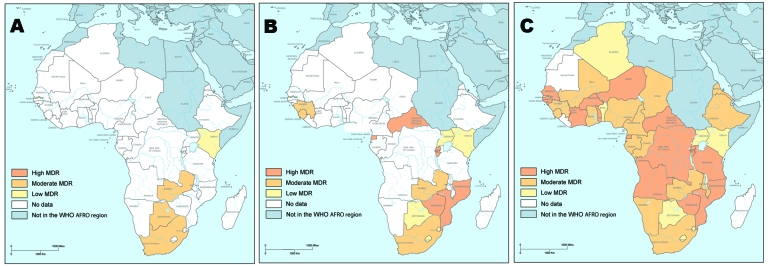
Prevalence of multidrug resistance (MDR) in Africa among combined tuberculosis cases. A) Data collected from the Third Global Report on Anti-tuberculosis Drug Resistance in the World of the World Health Organization (WHO) published in 2004 (*40*). B) Data from various recent WHO publications, peer-reviewed journal articles, and WHO’s Fourth Global Report (*1*). C) Formulaic estimates of Zignol et al. (*11*). AFRO, WHO Regional Office for Africa.

Panel B of the Figure includes data from various recent WHO publications, peer-reviewed journal articles, and the Fourth Global Report published in 2008 (*1*). Indications of geographic clustering are apparent; countries with a high prevalence of MDR TB are clustered in the southeastern and central regions of Africa. Panel B of the Figure also shows the 11 countries that have MDR TB rates >2.0 of all combined TB cases, including Democratic Republic of Congo, Rwanda, Equatorial Guinea, and Senegal, which were not included the WHO Third Global Report.

Panel C of the Figure includes formulaic estimates. On the basis of these estimates, high levels of MDR TB are not only located in the southeastern and central regions, but also in West Africa, which differs from results of the WHO Third Global Report.

### Effect of Year of Introduction of Rifampin

Dates of rifampin introduction were available for 11 countries (Table 3). Dates of introduction are clustered around the mid-1980s. The earliest and latest known introductions of rifampin were 1979 in South Africa and 1989 in Zambia. Zambia has a middle-level rate of MDR TB, South Africa has a high rate, and other countries have low rates. No clear association between date of rifampin introduction and MDR rates could be discerned.

**Table 3:** Years of introduction of rifampin in 11 African countries

Country	Date
Algeria	1980
Benin	1986
Botswana	1986
Côte d’Ivoire	1985
Gambia	1986
Malawi	1986
Mozambique	1986
Senegal	1986
South Africa	1979
Tanzania	1982
Zambia	1989

### Indicators for Continuous MDR Rates

Presurvey independent variables were evaluated through 3 types of linear regression analysis: correlation (Table 4), univariate, and multivariate regression (data not shown). Regarding associations with MDR rates, correlation analysis showed a significant association with retreatment failure rate (p = 0.043, r^2^ = 0.119). Several other univariate and multivariate linear models, such as treatment failure × retreatment failure and case detection × treatment failure × retreatment failure, were marginally significant. However, no other models that used presurvey data were more predictive than retreatment failure rate alone. For postsurvey variables, univariate analysis (Table 4) also showed a statistically significant association between MDR rates and retreatment failure rates.

**Table 4:** Correlation analysis between country-level TB-related indicators and rates of MDR TB in African countries*

Factor	Average, presurvey years			Average, postsurvey years	
	Coefficient	p value†		Coefficient	p value†
Incidence rate/100,000/y	0.09	0.592		0.03	0.865
Case detection rate (new ss+)	–0.14	0.380		–0.10	0.585
Treatment indicators					
Cured	0.03	0.842		0.18	0.330
Completed	–0.13	0.446		–0.21	0.256
Died	–0.10	0.542		0.04	0.840
Failed	0.12	0.498		0.17	0.341
Defaulted	–0.07	0.664		–0.05	0.781
Succeeded	–0.08	0.653		0.08	0.676
Retreatment indicators					
Cured	–0.03	0.881		0.08	0.674
Completed	–0.20	0.258		–0.24	0.217
Died	–0.06	0.728		–0.11	0.588
Failed	0.34	0.043‡		0.41	0.029‡
Defaulted	0.04	0.798		0.12	0.540
Succeeded	–0.16	0.351		–0.07	0.728
Year 2005 only variables					
Prevalence/100,000				0.14	0.404
Mortality rate/100,000/y				0.02	0.894
HIV/TB co-infection (%)				–0.09	0.593
Male/female ratio: case notifications				–0.05	0.787
Health expenditures (US $ per capita)				–0.01	0.956

*TB, tuberculosis; MDR TB, multidrug-resistant TB; ss+, sputum sample positive. †Marginally statistically significant trend (p<0.10). ‡Statistically significant trend (p<0.05).

### Comparison of Presurvey and Postsurvey Indicator Averages

Using the Wilcoxon signed rank test, we determined that 5 of 14 indicators showed a significant (p<0.05) change between presurvey and postsurvey years. Treatment default showed a decrease; incidence rate, treatment cured, treatment success, and retreatment death rate increased between time intervals.

### Indicators for MDR TB Categories

When considered through nonparametric trend analysis, there was a marginally significant positive association between MDR TB categories and retreatment failure rates (data not shown). Median retreatment failure rates were higher within relatively higher categorized MDR TB countries.

## Discussion

Our visual mapping indicates that MDR TB is likely to be more prevalent in Africa than previous reports indicated. The latest WHO Global Report on Anti-tuberculosis Drug Resistance in the World published in March 2008 (*1*) indicated rates of drug-resistant TB in Africa to be among the lowest worldwide. However, as of April 2008, 25 of the 46 countries in the AFRO region still had not completed a national study to investigate levels of MDR TB. By incorporating the latest national studies on MDR TB in Africa, and classifying countries on the basis of MDR TB rates in combined cases of TB, we found worrisome trends: 6 countries that were not included in the WHO Third Global Report published in 2004 have MDR TB rates >2.0% of all combined TB cases. This finding suggests that completing DRSs for all or most countries in the AFRO region is urgently needed and that the MDR TB threat in Africa could be much higher than originally assessed by WHO in its previous report in 2004. Drug-resistant strains, along with HIV/AIDS, are causing the biggest challenge to efficient management and control of TB.

The lower rates of MDR TB in Africa, when compared with rates in Eastern Europe or South America, could be related to the fact that for many years Africa was neglected and TB was not treated. Alternatively, later introduction of rifampin in drug regimens is often cited as an explanation for these low rates. However, for the few countries for which data were available, we did not find a statistically significant relationship between year of rifampin introduction and level of MDR TB.

We subsequently set out to investigate linear associations between country-specific factors as well as categorical trends in TB management to discriminate levels of MDR TB in Africa. Retreatment failure rate was the most predictive indicator of MDR TB rates; other variables such as average case detection rate (1995–2005), average TB incidence rate (1995–2005), TB prevalence (2005), and HIV/TB co-infection (2005) did not show a linear relationship. Categorical analysis showed similar results.

One can speculate about the reasons that retreatment failure rates may be associated with higher rates of MDR TB. The relationship may simply be an association without causation, i.e., that retreatment fails because a given patient may already have MDR TB. In that sense, retreatment failure is simply a marker for preexisting MDR TB. However, it is also possible that retreatment may be a cause of MDR TB. Current WHO recommendations for retreatment regimens could lead to development of MDR TB or XDR TB in many instances because suggested retreatment regimens potentially amount to addition of 1 drug to a failing regimen, thereby intensifying the level of drug resistance.

We subsequently investigated whether combining the retreatment failure rate with other indicators in our model could generate a predictive combination for MDR TB rates. Whereas the univariate linear and the multivariate regression model showed a marginally significant association with either treatment failure, case detection rate, or both, retreatment failure as a sole indicator remained the most significant. This finding may indicate that, regardless of the successful record of a given NTP in detecting cases and subsequently successfully treating them with a category I regimen, the highest MDR TB rates were found in the countries with the highest retreatment failure rates (category II).

We also investigated levels of variables reported in years after a DRS to determine whether the result of the survey affected these variables. Five of the 14 variables measured showed a statistically significant change over time: 4 variables (case detection rates, treatment cure rates, treatment success rates, and retreatment death rates) increased after the survey, and treatment defaulters decreased. Unfortunately, we cannot determine the effect of those 5 indicators on MDR TB rates because most countries surveyed reported only 1 DRS. Retreatment failure, which was shown to be the most predictive indicator of MDR TB rates, did not change over time. This finding suggests that MDR TB rates have increased in the years after the survey. To draw a parallel with physics, if the acceleration of an object remains constant over time, its speed increases. Therefore, it seems critical for countries who have already reported DRS results to undergo a second DRS to monitor the evolution of the MDR TB rate originally reported and help determine the effect of newly installed healthcare and TB treatment systems on drug resistance. For example, the only DRS reported from Kenya (conducted in 1995) reported an MDR TB rate of 0.0%. In 2008, the validity of this result is highly questionable given the recently published high MDR TB rates of neighboring countries (Figure).

This research was limited by the differing country-level data estimates and the ecologic study design. Twenty one of the 39 countries analyzed have TB estimates from WHO or peer-reviewed journals. However, the other 19 countries required formulaic estimates of MDR TB rates. The effect of these differing estimation methods depends on the congruity of future country-level estimates relative to the figures accepted in this study. Therefore, analyses should be repeated as more definitive estimates become available. Second, the ecologic study design, which analyzes these TB-related factors on a population level, limits the transference of any apparent relationships to the individual level. However, evidence shows that an effective DOTS program can limit the development of drug resistance on the individual TB patient level (*41*). Lastly, the small sample size of 39 countries limits the power to statistically determine differences. As a final note, this report hoped to realize 2 goals: to provide a composite of MDR TB in Africa and to identify relationships and trends.

Trends and relationships aside, MDR TB in Africa is a burgeoning obstacle that shows no signs of regression. The prevalence of MDR TB remains below the levels seen in Central Europe and parts of Latin America. However, in Africa, the tragically high HIV prevalence and limited funds and infrastructure dedicated to healthcare are serious factors. As the outbreak of MDR TB/HIV co-infection in New York City in the mid-1990s taught the medical community, this co-infection is pernicious, difficult, and overwhelmingly expensive to treat. This situation begs the question: if the continent is finding difficulty addressing TB, a well-defined disease caused by a well-defined agent, which is fully treatable with effective and affordable drugs through internationally recommended guidelines, then how will the continent fare against MDR TB and XDR TB?
